# Pre-ablation levels of brain natriuretic peptide are independently associated with the recurrence of atrial fibrillation after radiofrequency catheter ablation in patients with nonvalvular atrial fibrillation

**DOI:** 10.1007/s00380-018-1267-5

**Published:** 2018-09-20

**Authors:** Junichiro Miake, Masaru Kato, Kazuyoshi Ogura, Kazuhiko Iitsuka, Akihiro Okamura, Takuya Tomomori, Daiki Tsujimoto, Masahiko Kato, Kazuhiro Yamamoto

**Affiliations:** 1 Division of Molecular Pharmacology, Department of Pathophysiological and Therapeutic Science, Faculty of Medicine, Tottori University, Yonago, 683-8503 Japan; 2 Division of Cardiovascular Medicine, Endocrinology and Metabolism, Department of Molecular Medicine and Therapeutics, Faculty of Medicine, Tottori University, Yonago, 683-8503 Japan

**Keywords:** Atrial fibrillation, Catheter ablation, Recurrence, Biomarkers, Brain natriuretic peptide

## Abstract

Association between pre-ablation levels of biomarkers of cardiac and endothelial dysfunctions, CHADS2, CHA2DS2-VASc, and APPLE scores and the recurrence of atrial fibrillation (AF) after radiofrequency catheter ablation has not been fully studied. A total of 254 patients with nonvalvular AF were prospectively followed for AF recurrence after a single ablation procedure. During a two-year follow-up period, AF recurred in 65 (25.6%) patients. Patients with AF recurrence had significantly greater baseline ln brain natriuretic peptide (BNP) than those without AF recurrence (*P* < 0.01), whereas there were no significant differences in the levels of biomarkers of endothelial dysfunction and points of scoring systems. In the Cox regression analyses, the baseline ln BNP was significantly independently associated with AF recurrence (adjusted HR =1.286, 95% CI =1.000–1.655, *P* < 0.05). The baseline levels of ln BNP were significantly associated with rhythm at blood collection, age, sex, and left atrial diameter, and left ventricular ejection fraction (*P* < 0.05).The subgroup analysis showed a significant interaction on the risk of AF recurrence between ln BNP, sex difference, and rhythm at blood collection (*P* for interaction < 0.05). In conclusion, the results suggest that the pre-ablation levels of ln BNP are useful to evaluate the risk of AF recurrence after ablation therapy; however, there is a need to be careful while using BNP as a biomarker for the risk of AF recurrence by taking account of the effects of rhythm status at blood collection and sex difference.

## Introduction

Atrial fibrillation (AF), one of the commonest arrhythmias [1], is associated with an increased morbidity and mortality [2,3]. Haïssaguerre et al. have reported that the pulmonary veins are the dominant sources of triggers for initiating AF in most patients [4]. Catheter ablation of AF with isolation of the pulmonary veins, which has become the standard therapy for symptomatic patients with AF that is refractory to medical therapy [5], is highly effective at curing paroxysmal or persistent AF [6]. However, AF recurs after ablation therapy in some patients and the risk of such recurrence has been difficult to predict [7].

Inflammatory, oxidative stress [8–10] and atrial remodeling [11,12] have been reported as the etiology of AF development. In addition, it has been reported that multiple risk scores and biomarkers considering these etiologies may be predictors of recurrence after atrial fibrillation ablation treatment. The CHADS_2_ and CHA_2_DS_2_-VASc scores, both of which comprise common cardiovascular risk factors, has been originally developed for predicting stroke and thromboembolism [13]. These scores have been reported to be associated with AF recurrence in patients with paroxysmal AF after a single ablation procedure [14], and with long-term outcomes in patients with paroxysmal AF and persistent AF after AF ablation [15]. However, studies on the predictive value of the CHADS_2_ and CHA_2_DS_2_-VASc scores on the recurrence of AF after ablation had inconsistent results [16]. Then, the APPLE score has been developed to predict AF recurrence after an ablation procedure in patients with paroxysmal and persistent AF. This score comprises five clinical variables such as age, type of AF, renal function, and echocardiographic variables [17]. The APPLE had better prediction of AF recurrence between 3 and 12 months after catheter ablation than CHADS_2_ and CHA_2_DS_2_-VASc scores [17]. Of biomarkers, brain natriuretic peptide (BNP), an intensively studied biomarker of cardiac dysfunction, has been reported being a predictor of the recurrence of AF [18,19]. Biomarkers of endothelial dysfunction caused by inflammatory, oxidative stress, such as inflammation-related leukocyte adhesion molecules (vascular cell adhesion molecule-1 [VCAM1]; intercellular adhesion molecule-1 [ICAM1]; and endothelial cell-leukocyte adhesion molecules-1 [ELAM1]), a physiological endothelial anticoagulant (thrombomodulin [TM]), and a platelet adhesion molecule (von Willebrand factor [VWF]) have been reported to be associated with risk of incident AF [20–24]. However, there were significant heterogeneities between studies to test associations between BNP and the recurrence of AF in meta-analysis studies, and such heterogeneities have not been fully explained [18,19]. Moreover, associations between biomarkers of endothelial dysfunction and the recurrence of AF have not been fully studied. Furthermore, associations of the risk scores, biomarkers and atrial remodeling with the recurrence of AF have not been deeply and systematically studied.

In this study, we measured pre-ablation levels of biomarkers of cardiac and endothelial dysfunction, and evaluated scores of CHADS_2_ and CHA_2_DS_2_-VASc, and APPLE scoring systems in patients with nonvalvular paroxysmal or persistent AF. We then examined associations between these pre-ablation variables and the recurrence of AF after radiofrequency catheter ablation. Moreover, a variable significantly and independently associated with the recurrence of AF was tested for associations with a number of key risk variables such as age, sex, type of AF, history of heart failure, history of hypertension, history of diabetes mellitus, history of stroke or transient ischemic attack, history of coronary or peripheral vascular disease, renal function, LAD, and LVEF [25]. Finally, we performed subgroup analyses and tested interactions for a baseline variable significantly associated with the recurrence of AF.

## Methods

### Study cohort and design

Consecutive patients with nonvalvular paroxysmal or persistent AF who underwent a single catheter ablation from March 2013 to December 2015 in Tottori University Hospital, Japan were enrolled and followed prospectively for the recurrence of AF after ablation. Paroxysmal AF was defined as self-terminating AF lasting for up to 7 days or AF episodes cardioverted within 7 days, and persistent AF as AF lasting between 7 days and 1 year or AF episodes terminated by cardioversion either with drugs or by direct current cardioversion after 7 days or more [5]. Levels of estimated glomerular filtration rate (eGFR) were calculated with the following equation established by the Japanese Society of Nephrology: eGFR (mL/min/1.73 m^2^)  = 194 × serum creatinine level^−1.094^ × age^−0.287^ (× 0.739 if female).[3] The CHADS_2_ score comprised congestive heart failure, hypertension, age ≥ 75, diabetes mellitus, and stroke/transient ischemic attack and the CHA_2_DS_2_-VASc score comprised congestive heart failure, hypertension, age ≥ 75 years, diabetes mellitus, stroke/transient ischemic attack, vascular disease, age 65–74 years, and female sex [13]. The APPLE score comprised age > 65 years, persistent AF, reduced eGFR < 60 ml/min/1.73 m^2^, left atrial diameter (LAD) ≥ 43 mm, and left ventricular ejection fraction (LVEF) < 50%. The APPLE had better prediction of AF recurrence between 3 and 12 months after catheter ablation than CHADS_2_ and CHA_2_DS_2_-VASc scores [17]. Patients with one or more of the following were excluded from candidates for ablation of AF in this study: age less than 20 years or over 85 years; cerebral or myocardial infarction within 6 months of onset; uncontrolled heart failure or left ventricular ejection fraction < 40%; severe chronic obstructive pulmonary disease; contraindications to anticoagulants; thrombus formation within the left atrial appendage; LAD > 60 mm; moderate or severe valvular insufficiency or stenosis; connective tissue disease; and active cancer. It was also planned to exclude patients with hypertrophic/dilated cardiomyopathy or old myocardial infarction, end-stage renal disease or on hemodialysis: five patients with hypertrophic cardiomyopathy and six with end-stage renal disease or on hemodialysis were thus excluded. This study was approved by the Ethics Committee of Faculty of Medicine, Tottori University, and conducted in compliance with the ethical principles of the Declaration of Helsinki. Written informed consent was obtained from all individual participants included in the study.

### Catheter ablation protocol

The protocol for catheter ablation of AF has been reported previously [11]. Briefly, prior to the ablation procedure, all patients received effective oral anticoagulation therapy for at least four weeks and underwent transesophageal echocardiography and enhanced cardiac computed tomography for 3D mapping. Antiarrhythmic drugs were discontinued for more than five times their half-lives. Oral anticoagulants were discontinued before the procedure: direct oral anticoagulants for one day and warfarin for five days, and replaced with unfractionated heparin until 6 h prior to the ablation procedure. Bipolar electrograms were continuously recorded on a digital recording system (Labsystem PRO; Bard Electrophysiology, Lowell, MA, USA). A 20-pole three-sited mapping catheter (BeeAT; Japan Lifeline, Tokyo, Japan) was used to record bipolar electrograms of the superior vena cava, right atrium, and coronary sinus. After transseptal puncture, heparin was continuously infused to maintain an activated clotting time of 300–400 s. Two decapolar circular mapping catheters (Lasso; Biosense Webster, Diamond Bar, CA, USA) were located in the pulmonary veins. A 3.5 mm irrigated-tip ablation catheter (Thermocool; Biosense Webster) was advanced into the LA to achieve bilateral circumferential pulmonary vein isolation with the endpoint of bidirectional conduction block between the LA and pulmonary vein. A point-by-point radiofrequency current was delivered for 30 s with a power of up to 40 watts and a target temperature less than 43°C using a 3D mapping system (CARTO3; Biosense Webster), whereas the power was limited to 20 watts at the site close to the esophagus. After achieving pulmonary vein isolation, a bidirectional conduction blocking line was created at the cavotricuspid isthmus in all patients. The ablation procedure was performed under conscious sedation with continuous monitoring of blood pressure and oxygen saturation.

### Blood sample collection and processing

Blood was sampled from the femoral vein (FV) with an 18 gauge needle prior to the ablation procedure on the day of ablation therapy, placed immediately on ice and centrifuged at 3,000*g* for 15 min at 4°C. The resultant serum or plasma was aliquoted to different tubes to avoid repeated freeze–thaw cycles and stored at − 80°C till use. Serum soluble ELAM1 (sELAM1; ng/mL), soluble VCAM1 (sVCAM1; ng/mL), and soluble ICAM1 (sICAM1; ng/mL) were measured by enzyme-linked immunosorbent assay (ELISA; R&D systems, Minneapolis, MN, USA); plasma VWF activity (%) by latex agglutination immunoassay (LAIA; Siemens Healthcare Diagnostics Products GmbH, Marburg, Germany); serum soluble TM (sTM; FU/mL) by ELISA (Kyowa Pharma Chemical, Takaoka, Toyama, Japan); plasma BNP (pg/mL) by chemiluminescent enzyme immunoassay (CLEIA; Fujirebio, Shinjuku, Tokyo, Japan). The within-run reproducibility was < 8% for ELISAs and CLEIA and < 20% for LAIA. All assays were performed by investigators who were blinded to all clinical information about the participants.

### Clinical outcomes and follow-up

The primary clinical outcome was recurrent AF lasting over 30 s off antiarrhythmic drugs. Episodes during the blanking period of the first 90 days after the ablation procedure were not considered as recurrences [5]. All patients underwent continuous ECG monitoring for 3 days following the procedure until discharge. Follow-up checks were performed 1, 2, 3, 6, 9, 12, 16, 20, and 24 months after the ablation procedure and comprised physical examination, 12-lead electrocardiogram, and 24-h Holter monitoring. Patients were instructed to self-monitor their pulses daily and report any irregularity of pulse immediately, at which point event monitoring or 14-day Holter monitoring was performed. Oral anticoagulant therapy was continued in all patients with CHA_2_DS_2_-VASc scores of two or more points after the procedure, but discontinued at 6 months in those whose scores were less than 2 points.

### Statistical analysis

Categorical data are presented as frequency (percentage [%]) and were compared with Fisher’s exact test. Continuous data are presented as mean (standard deviation [SD]) or as median (interquartile range) for skewed distributions. Normality was tested with the Kolmogorov–Smirnov test. Normally distributed continuous variables were compared using the independent Student’s *t* test and skewed data using the non-parametric Mann–Whitney *U* test. Analyses were performed after natural logarithmic transformation (ln) of skewed biomarkers. Freedom from AF was reported as crude event rates and by means of a time-to-event analysis using the Kaplan–Meier method. Variables with *P* value < 0.10 in the group comparison were further evaluated by the Cox proportional hazards regression analyses to determine their associations with the recurrence of AF. The variables with *P* value < 0.10 in the univariate Cox proportional hazards regression analysis were further evaluated by the multivariate Cox proportional hazards regression analysis. Multiple regression analysis via the forced entry procedure was performed to test correlations of a normally distributed continuous variable with other variables. Statistical analyses were performed using R software (version 3.4.0; R Foundation for Statistical Computing, Vienna, Austria), and *P* value < 0.05 was considered to denote statistical significance.

## Results

### Patient characteristics in patients with and without the recurrence of AF

In total, 254 patients with AF (mean age of 65.5 [SD =9.3]; 68.9% paroxysmal and 31.1% persistent; all first session) were included in this study (Table 1). All ablation procedures were successful. The median length of follow-up for censored cases was 730 days. AF recurred in 65 (25.6%) patients during follow-up. Ln BNP was significantly greater in patients with the recurrence of AF than those without the recurrence of AF (*P* = 0.006) and was an only variable which significantly differed between the groups (Table 1). Although statistically not significant, uses of β-blocker and loop diuretics were more frequent and ln sTM were lower in patients with the recurrence of AF than those without the recurrence of AF (*P* value < 0.10, Table 1).

**Table 1:** Baseline characteristics of the study patients

Variable	All patients (*n* = 254)	Non-recurrence (*n* = 189)	Recurrence (*n* = 65)	*P* value
Age, years	65.5 (9.3)	66.0 (9.2)	64.0 (9.4)	0.141
Type of AF				0.438
Paroxysmal AF, *n* (%)	175 (68.9)	133 (70.4)	42 (64.6)	
Persistent AF, *n* (%)	79 (31.1)	56 (29.6)	23 (35.4)	
Sex, male, *n* (%)	184 (72.4)	139 (73.5)	45 (69.2)	0.522
BMI, kg/m^2^	23.6 (3.0)	23.5 (3.0)	24.0 (3.2)	0.207
Mean blood pressure, mmHg	92.9 (13.0)	92.9 (12.5)	93.0 (14.4)	0.948
Rhythm at blood sampling				0.106
SR, *n* (%)	152 (59.8)	119 (63.0)	33 (50.8)	
AF, *n* (%)	102 (40.2)	70 (37.0)	32 (49.2)	
Current smoker, *n* (%)	21 (8.3)	16 (8.5)	5 (7.7)	1.000
CHADS_2_ score	1.0 (1.0–2.0)	1.0 (1.0–2.0)	1.0 (1.0–2.0)	0.170
CHA_2_DS_2_-VASc score	2.0 (1.0–3.0)	2.0 (1.0–3.0)	2.0 (1.0–3.0)	0.271
APPLE score	1.0 (1.0–2.0)	1.0 (1.0–2.0)	2.0 (1.0–3.0)	0.481
History of HF, *n* (%)	34 (13.4)	22 (11.6)	12 (18.5)	0.204
History of HT, *n* (%)	144 (56.7)	110 (58.2)	34 (52.3)	0.469
History of DM, *n* (%)	32 (12.6)	27 (14.3)	5 (7.7)	0.198
History of stroke/TIA, *n* (%)	23 (9.1)	19 (10.1)	4 (6.2)	0.456
History of vascular disease, *n* (%)	13 (5.1)	10 (5.3)	3 (4.6)	1.000
eGFR, mL/min/1.73 m^2^	80.4 (21.7)	80.4 (20.9)	80.3 (24.1)	0.986
LAD, mm	39.9 (6.8)	39.7 (6.5)	40.6 (7.7)	0.357
LVEF, %	62.3 (9.1)	62.4 (9.3)	62.0 (8.8)	0.730
Medications				
Type of anticoagulants				0.273
VKA, *n* (%)	48 (18.9)	39 (20.6)	9 (13.8)	
DOAC, *n* (%)	206 (81.1)	150 (79.4)	56 (86.2)	
Acetylsalicylic acid, *n* (%)	16 (6.3)	12 (6.3)	4 (6.2)	1.000
ACEI/ARB, *n* (%)	104 (40.9)	80 (42.3)	24 (36.9)	0.468
β-blocker, *n* (%)	84 (33.1)	56 (29.6)	28 (43.1)	0.066
CCB, *n* (%)	83 (32.7)	64 (33.9)	19 (29.2)	0.542
Digitalis, *n* (%)	12 (4.7)	9 (4.8)	3 (4.6)	1.000
Loop diuretics, *n* (%)	35 (13.8)	22 (11.6)	13 (20.0)	0.099
Aldosterone antagonist, *n* (%)	14 (5.5)	9 (4.8)	5 (7.7)	0.358
Statin, *n* (%)	53 (20.9)	40 (21.2)	13 (20.0)	1.000
NCB, *n* (%)	100 (39.4)	69 (36.5)	31 (47.7)	0.141
Bepridil, *n* (%)	20 (7.9)	13 (6.9)	7 (10.8)	0.299
Amiodarone, *n* (%)	11 (4.3)	7 (3.7)	4 (6.2)	0.480
ln BNP	4.3 (1.1)	4.2 (1.1)	4.6 (1.0)	0.006
ln sTM	0.95 (0.27)	0.97 (0.28)	0.90 (0.26)	0.079
ln sELAM1	3.3 (0.4)	3.3 (0.4)	3.3 (0.5)	0.870
ln sICAM1	5.1 (0.4)	5.1 (0.4)	5.2 (0.4)	0.162
ln sVCAM1	6.7 (0.3)	6.7 (0.3)	6.7 (0.3)	0.363
VWF activity, %	166.3 (77.4)	170.4 (77.2)	154.4 (77.1)	0.151

The values are presented as number (%), mean (standard deviation), or median (interquartile range)

*ACE* angiotensin converting enzyme, *AF* atrial fibrillation, *ARB* angiotensin receptor blocker, *BMI* body mass index, *BNP* brain natriuretic peptide, *CCB* calcium channel blocker, *DM* diabetes mellitus, *DOAC* direct oral anticoagulant, *eGFR* estimated glomerular filtration rate, *HF* heart failure, *HT* hypertension, *LAD* left atrial diameter, *LVEF* left ventricular ejection fraction, *NCB* natrium channel blocker, *sELAM1* soluble endothelial cell-leukocyte adhesion molecules-1, *sICAM1* soluble intercellular adhesion molecule-1, *SR* sinus rhythm; sTM = soluble thrombomodulin; sVCAM1 = soluble vascular cell adhesion molecule-1, *TIA* transient ischemic attack, *VKA* vitamin K antagonist, *VWF* von Willebrand factor

**Fig. 1:**
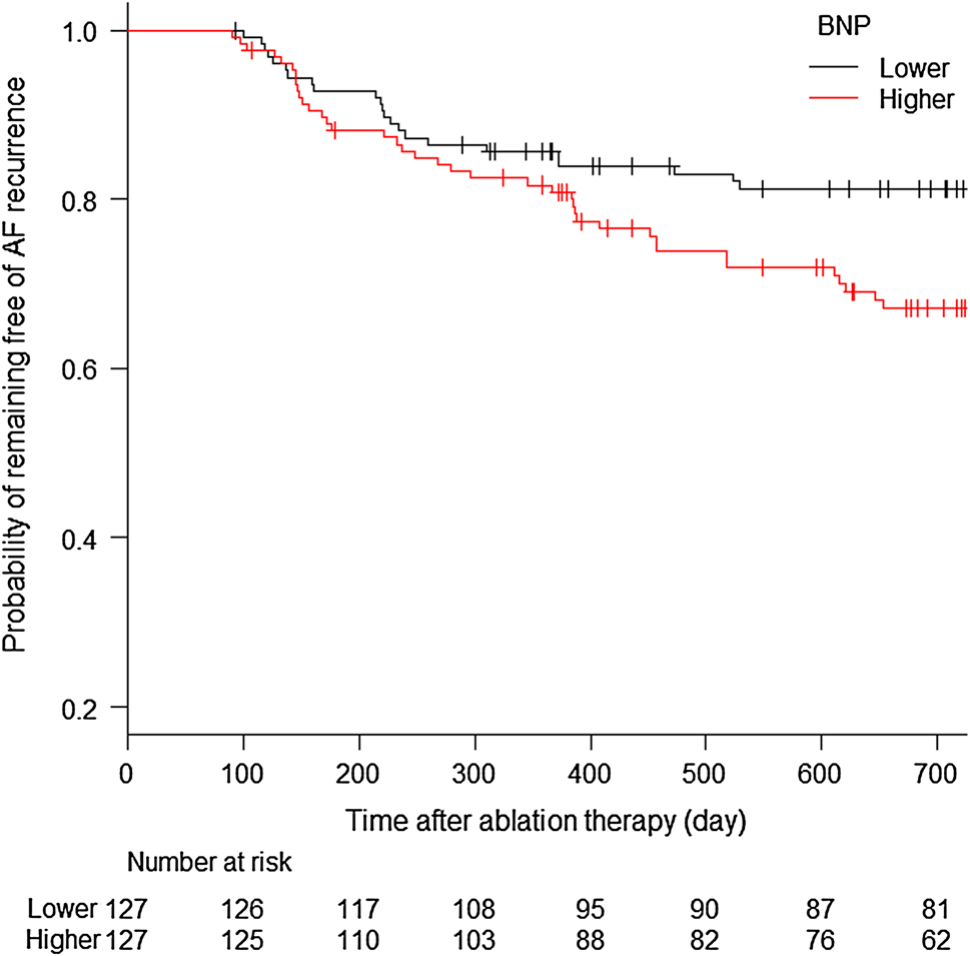
Probability of remaining free of the recurrence of AF in patients with the lower and higher levels of BNP. Patients were divided into two groups by the median concentration of BNP. Patient with lower level of BNP had a greater probability of remaining free of the recurrence of AF than those with higher level of BNP (*P* value by log-rank test = 0.011). Two-year recurrence-free survival rates were 0.812 (95% CI = 0.730–0.871) and 0.672 (95% CI = 0.578–0.749) in patients with the lower and higher levels of BNP, respectively. *BNP *brain natriuretic peptide

Because baseline ln BNP was significantly greater in patients with the recurrence of AF than those without the recurrence of AF, the study patients were divided into two groups by the median concentration of BNP (groups with lower and higher levels of BNP) and then a probability of remaining free of the recurrence of AF after ablation procedure with a three-month blanking period was evaluated in the two groups. As a result, patient with the lower level of BNP had a greater probability of remaining free of the recurrence of AF than those with the higher level of BNP (*P* value by log-rank test = 0.011) as shown in Fig. 1. Two-year recurrence–free survival rates were 0.812 (95% confidence interval [CI] =  0.730–0.871) and 0.672 (95% CI = 0.578–0.749) in patients with the lower and higher levels of BNP, respectively.

### Associations of baseline variables with the recurrence of AF

To determine associations of baseline variables with the recurrence of AF, the univariate and multivariate Cox proportional hazards regression analyses. In the univariate Cox proportional hazards regression analyses, only ln BNP was significantly associated with the recurrence of AF (unadjusted HR = 1.353, 96% CI = 1.068–1.713, *P* = 0.012), whereas use of β-blocker, use of loop diuretics, and ln sTM were not significantly associated with the recurrence of AF (unadjusted HR = 1.582, 96% CI = 0.968–2.585, *P* = 0.067; unadjusted HR = 1.603, 96% CI = 0.873–2.945, *P* = 0.128; unadjusted HR = 0.447, 96% CI = 0.180–1.107, *P* = 0.082, respectively) as shown in Table 2. In the multivariate Cox proportional hazards regression analysis, ln BNP remained significantly associated with the recurrence of AF (adjusted HR = 1.286, 96% CI = 1.000–1.655, *P* = 0.049) when tested with the use of β-blocker and ln sTM, *P* values of which were < 0.10 in the univariate Cox regression analysis (Table 2).

**Table 2:** Univariate and multivariate Cox regression analyses

Variable	Univariate			Multivariate		
	Unadjusted HR	95% CI	*P* value	Adjusted HR	95% CI	*P* value
β-blocker (use = 1)	1.582	0.968–2.585	0.067	1.345	0.804–2.251	0.258
Loop diuretics (use = 1)	1.603	0.873–2.945	0.128	not selected		
ln BNP (per 1 increase)	1.353	1.068–1.713	0.012	1.2860	1.000–1.655	0.049
ln sTM (per 1 increase)	0.447	0.180–1.107	0.082	0.4960	0.207–1.190	0.116

After performing univariate Cox regression analysis, variables with *P* value < 0.10 were further evaluated by multivariate Cox regression analysis

*BNP* brain natriuretic peptide, *CI* confidence interval, *HR* hazard ratio, *ln* natural logarithm, *sTM* soluble thrombomodulin

### Correlations between ln BNP and the key risk variables

Because ln BNP remained significantly and independently associated with the recurrence of AF, we performed a multiple regression analysis to test correlations between ln BNP and the key risk variables by the forced entry procedure. In a multivariate regression model controlling simultaneously for rhythm at blood collection, age, sex, type of AF, body mass index (BMI), eGFR, LAD, and LVEF, rhythm at blood collection, age, sex, LAD, and LVEF were significantly associated with the level of ln BNP (Table 3): specifically, AF at blood collection was significantly associated with the substantially increased level of ln BNP (adjusted β = 0.482, 95% CI = 0.213–0.751, *P* value < 0.001).

**Table 3:** Linear regression analyses for ln BNP

Variables	Single regression		Multiple regression	
	Unadjusted β coefficient	*P* value	Adjusted β coefficient	*P* value
Rhythm at blood collection (AF = 1, SR =0)	0.857 (0.613 to 1.101)	< 0.001	0.482 (0.213 to 0.751)	< 0.001
Age (per year increase)	0.036 (0.023 to 0.050)	< 0.001	0.027 (0.015 to 0.040)	< 0.001
Sex (male = 1, female = 0)	− 0.284 (− 0.574 to 0.005)	0.054	− 0.438 (− 0.722 to − 0.154)	0.003
Type of AF (persistent AF = 1, paroxysmal AF = 0)	0.877 (0.617to 1.137)	< 0.001	0.125 (− 0.180 to 0.430)	0.420
BMI (per kg/m^2^ increase)	0.007 (− 0.036 to 0.050)	0.752	− 0.035 (− 0.072 to 0.001)	0.058
eGFR (per mL/min/1.73 m^2^ increase)	− 0.007 (− 0.013 to − 0.001)	0.015	− 0.004 (− 0.009 to 0.002)	0.226
LAD (per mm increase)	0.074 (0.057 to 0.091)	< 0.001	0.049 (0.030 to 0.068)	<0.001
LVEF (per % increase)	− 0.031 (− 0.045 to − 0.017)	< 0.001	− 0.023 (− 0.035 to − 0.011)	<0.001

Abbreviations are shown as in Table 1. In a multiple regression analysis, the regression coefficient estimate (β) was adjusted for all other variables listed.

### Subgroup analyses of risks of the recurrence of AF for ln BNP

We further performed subgroup analyses of risks of the recurrence of AF for ln BNP by dividing patients with the risk variables and the interaction effects of ln BNP with other risk variables, specifically with various combinations of rhythm status at blood collection and other risk variables because rhythm status at blood collection was significantly associated with substantially different levels of ln BNP (Table 3). Consequently, there was a significant interaction of ln BNP with sex and rhythm at blood collection (*P* for interaction = 0.011) as shown in Table 4: higher ln BNP was significantly associated with an increased risk of the recurrence of AF in a subgroup of male sex with sinus rhythm at blood collection (HR = 1.536, 95% CI = 1.095–2.154, *P* value = 0.013), but not in the other subgroups.

**Table 4:** Subgroup analyses of hazard ratios of ln BNP for the recurrence of atrial fibrillation

Subgroup	Recurrence, *n* (%)	Hazard ratio (95% CI) of ln BNP	*P* value	*P* for interaction
Rhythm at blood collection				0.449
Sinus rhythm (*n* = 152)	33 (21.7%)	1.397 (1.022–1.911)	0.036	
Atrial fibrillation (*n* = 102)	32 (31.4%)	1.153 (0.737–1.806)	0.533	
Age				0.328
< 65 (*n* = 105)	29 (27.6%)	1.230 (0.872–1.734)	0.238	
≥ 65 (*n* = 149)	36 (24.2%)	1.567 (1.126–2.181)	0.008	
Sex				0.791
Male (*n* = 184)	45 (24.5%)	1.372 (1.036–1.818)	0.027	
Female (*n* = 70)	20 (28.6%)	1.270 (0.806–2.000)	0.303	
Type of AF				0.666
Paroxysmal AF (*n* = 175)	42 (24.0%)	1.415 (1.076–1.861)	0.013	
Persistent AF (*n* = 79)	23 (29.1%)	1.271 (0.710–2.275)	0.419	
BMI				0.128
< 25 kg/m^2^ (*n* = 172)	40 (23.3%)	1.204 (0.902–1.606)	0.207	
≥ 25 kg/m^2^ (*n* = 82)	25 (30.5%)	1.821 (1.143–2.898)	0.012	
eGFR				0.375
≥ 60 ml/min/1.78 m^2^ (*n* = 216)	55 (25.5%)	1.297 (0.999–1.684)	0.051	
< 60 ml/min/1.78 m^2^ (*n* = 38)	10 (26.3%)	1.790 (0.951–1.684)	0.071	
LAD				0.443
< 40 mm (*n* = 129)	28 (21.7%)	1.213 (0.855–1.721)	0.280	
≥ 40 mm (*n* = 125)	37 (29.6%)	1.467 (1.029–2.090)	0.034	
LVEF				0.863
≥ 50% (*n* = 227)	58 (25.6)	1.398 (1.086 1.800)	0.009	
< 50% (*n* = 27)	7 (25.9)	1.294 (0.431 3.880)	0.646	
Age and rhythm at blood collection				0.583
Age < 65 with SR (*n* = 61)	16 (26.2%)	1.228 (0.815–1.851)	0.326	
Age < 65 with AF (*n* = 44)	13 (29.5%)	1.341 (0.538–3.342)	0.529	
Age ≥ 65 with SR (*n* = 91)	17 (18.7%)	1.867 (1.156–3.015)	0.011	
Age ≥ 65 with AF (*n* = 58)	19 (32.8)	1.105 (0.635–1.922)	0.724	
Sex and rhythm at blood collection				0.011
Male with SR (*n* = 104)	25 (24.0%)	1.536 (1.095–2.154)	0.013	
Male with AF (*n* = 80)	20 (25.0%)	1.221 (0.671–2.223)	0.513	
Female with SR (*n* = 48)	8 (16.7%)	0.999 (0.432–2.311)	0.999	
Female with AF (*n* = 22)	12 (54.5%)	0.743 (0.330–1.675)	0.474	
Type of AF and rhythm at blood collection				0.436
Paroxysmal AF with SR (*n* = 141)	30 (21.3%)	1.391 (0.997–1.943)	0.052	
Paroxysmal AF with AF (*n* = 34)	12 (35.3)	1.162 (0.641–2.108)	0.621	
Persistent AF with SR (*n* = 11)	3 (27.3%)	1.920 (0.353–10.430)	0.450	
Persistent AF with AF (*n* = 68)	20 (29.4%)	1.187 (0.628–2.244)	0.597	
BMI and rhythm at blood collection				0.640
BMI < 25 kg/m^2^ mm with SR (*n* = 109)	23 (21.1%)	1.147 (0.791–1.664)	0.470	
BMI < 25 kg/m^2^ mm with AF (*n* = 63)	17 (27.0%)	1.225 (0.698–2.151)	0.479	
BMI ≥ 25 kg/m^2^ mm with SR (*n* = 43)	10 (23.2%)	2.269 (1.170–4.401)	0.015	
BMI ≥ 25 kg/m^2^ mm with AF (*n* = 39)	15 (38.5%)	1.010 (0.437–2.335)	0.982	
eGFR and rhythm at blood collection				0.472
eGFR ≥ 60 ml/min/1.78 m^2^ mm with SR (*n* = 130)	28 (21.5%)	1.230 (0.881–1.716)	0.225	
eGFR ≥ 60 ml/min/1.78 m^2^ mm with AF (*n* = 86)	27 (31.3%)	1.258 (0.742–2.131)	0.394	
eGFR < 60 ml/min/1.78 m^2^ mm with SR (*n* = 22)	5 (22.7%)	3.702 (1.135–12.080)	0.030	
eGFR < 60 ml/min/1.78 m^2^ mm with AF (*n* = 16)	5 (31.3%)	1.000 (0.281–3.557)	1.000	
LAD and rhythm at blood collection				0.374
LAD < 40 mm with SR (*n* = 100)	19 (19.0%)	1.034 (0.684–1.562)	0.874	
LAD < 40 mm with AF (*n* = 29)	9 (31.0%)	2.033 (0.599–6.904)	0.255	
LAD ≥ 40 mm with SR (*n* = 52)	14 (26.9%)	2.382 (1.305–4.348)	0.005	
LAD ≥ 40 mm with AF (*n* = 73)	23 (31.5%)	0.994 (0.590–1.675)	0.981	
LVEF and rhythm at blood collection				0.876
LVEF ≥ 50% mm with SR (*n* = 141)	30 (21.3)	1.394 (0.997–1.949)	0.052	
LVEF ≥ 50% mm with AF (*n* = 86)	28 (32.6)	1.230 (0.765–1.977)	0.392	
LVEF < 50% mm with SR (*n* = 11)	3 (27.3)	1.487 (0.353–6.257)	0.589	
LVEF < 50% mm with AF (*n* = 16)	4 (25.0)	0.933 (0.137–6.338)	0.944	

Abbreviations are shown as in Table 1.

## Discussion

In the present study, baseline ln BNP levels were significantly higher in patients with than without the recurrence of AF, whereas there were no significant differences in clinical and echocardiographic variables, points of risk scores, and levels of biomarkers of endothelial dysfunction between patients with and without the recurrence of AF. During a 2-year follow-up period, patients with the higher level of ln BNP had a significantly higher recurrent rate of AF than those with the low level of ln BNP. Moreover, baseline ln BNP was significantly and independently associated with the recurrence of AF in the univariate and multivariate Cox regression analyses. Furthermore, baseline ln BNP levels were significantly associated with rhythm at blood collection, age, sex, and LAD, and LVEF. Finally, the subgroup analyses showed a significant interaction of ln BNP with rhythm status at blood collection and sex difference.

### Baseline clinical variables and scoring systems in the recurrence of AF

Many observational studies have reported predictors of the recurrence of AF after catheter ablation including the key risk variables [25]. Persistent AF and LAD, both of which are related to atrial remodeling [11,12], are most consistently recognized as important baseline predictors for the recurrence of AF [25]. A study showed that the APPLE score for the prediction of rhythm outcomes after catheter ablation of AF, which comprises age, type of AF, renal function, LAD, and LVEF, had a better prediction of the recurrence of AF than CHADS_2_ and CHA_2_DS_2_-VASc scores [17]. However, in this study, above-mentioned baseline clinical variables, CHADS_2_, CHA_2_DS_2_-VASc, APPLE scores were not significantly different between patients with and without the recurrence of AF. Mean age of patients enrolled in this study was 65.5 years, whereas those of patients in other studies to identify predictors of late recurrence after AF ablation, to evaluate the values of CHADS_2_, CHA_2_DS_2_-VASc, and the APPLE scores were 57–61 years old [16,17,25]. Furthermore, BMI and LAD of patients in this study were smaller, a period of follow-up longer than those of patients in other studies, and races of patients differed between studies. Therefore, these differences between the current study and other studies may have caused inconsistent results of predictive values of baseline clinical variables and scoring systems for the recurrence of AF.

### Biomarkers in the recurrence of AF

Inflammation and oxidative stress have been reported as the etiology of AF development [8–10] and biomarkers of endothelial dysfunction caused by inflammatory, oxidative stress have been reported to be associated with risk of incident AF [20–24]; however, an association between pre-ablation concentrations of endothelial dysfunction biomarkers and the recurrence of AF has not yet been investigated. In the current study, we found no significant endothelial dysfunction biomarkers associated with risk of the recurrence of AF. It has been reported that the risk associated with endothelial dysfunction for arrhythmia recurrence following catheter ablation was age-dependent and was higher in younger than older patients with AF [23]. Therefore, it is possible that baseline concentrations of endothelial dysfunction biomarkers have predictive values for the recurrence of AF in younger patients than those enrolled in the current study. Further study is needed to confirm this possibility.

Associations between natriuretic peptide and the recurrence of AF have been intensively studied. Meta-analyses have shown that the recurrence of AF was associated with higher pre-ablation concentrations of BNP than non-recurrence although results of the studies were significantly heterogeneous; however, such heterogeneities have not been fully explained [18,19]. In the current study, we found that baseline ln BNP was significantly and independently associated with the recurrence of AF. We also found that AF at blood collection was significantly associated with the substantially increased level of ln BNP. Moreover, the subgroup analyses showed a significant interaction of ln BNP with sex and rhythm at blood collection, which may explain the heterogeneities between studies to test associations between BNP and the recurrence of AF [18,19]. Taken together, in patients with nonvalvular AF, baseline BNP was suggested as a useful biomarker for the risk of late recurrence of AF after ablation; however, there is a need to be careful while using BNP as a biomarker for the recurrence of AF by taking account of the effects of rhythm status at blood collection and sex difference.

### Study limitations

Several limitations of our study need to be considered. This study was performed in a single center, which may have resulted in a selection bias: indeed, BMI and LAD of patients in this study were smaller, and age was higher than those of patients in other studies. Asymptomatic episodes of AF may have been missed. We did not evaluate the responses of biomarkers to ablation therapy. We were unable to establish causal relationships between biomarker concentrations and the recurrence of AF because of a prospective observational study. However, our study also had several strengths, including a period of follow-up longer than other studies, deep and systematical evaluation of associations of the risk scores, biomarkers and atrial remodeling with the recurrence of AF, which allowed us to recognize the usefulness of BNP as a predictor of recurrence after ablation treatment of atrial fibrillation and at the same time to recognize the attention at that time.

## Conclusions

In this study, baseline levels of BNP, but not those of biomarkers of endothelial dysfunction or points of risk scoring systems, were independently associated with the recurrence of AF after radiofrequency catheter ablation. Baseline ln BNP levels were significantly associated with rhythm at blood collection, age, sex, and LAD, and LVEF, and the subgroup analyses showed significant interactions of ln BNP with sex, and rhythm at blood collection. These results suggest that pre-ablation levels of ln BNP are useful to evaluate the risk of AF recurrence after ablation; however, there is a need to be careful while using BNP as a biomarker for the risk of AF recurrence by taking account of the effects of rhythm status at blood collection and sex difference.
